# Semi-Annual Report of the Buffalo Hospital of the Sisters of Charity

**Published:** 1849-04

**Authors:** 


ART. II.—*Semi-Annual Report of the Buffalo Hospital of the Sisters of Charity.*

We insert with pleasure the following first semi-annual report of the only organized hospital at present in practical operation in this city. The reader will perceive that already, in the short period of its existence, much good has been effected, notwithstanding the accommodations and resources of the institution are as yet limited. The donations of benevolent persons here, and in other places, have exceeded immediate necessities, sufficiently for the commencement of additions to the building. We trust that farther aid may be obtained by private contributions, and by legislative appropriation, sufficient, not only to complete the improvements already in progress, but to undertake others which will extend still more the facilities for affording relief to the sick and destitute. As its title imports, the hospital is placed under the executive management of the Sisters of Charity. The sister superior, or, as she is called, the *sister servant*, has the direction of the interior affairs of the Institution. Associated with her, for ministration to the necessities and comfort of the sick, are, at present, four sisters of the order. The fact that the services of these intelligent, educated, and pious sisters are bestowed without compensation, conduces greatly to the economy of the Institution; but aside from this, the same capabilities and fidelity could not be purchased by any pecuniary consideration. No salary, however great, could afford a substitute for motives derived from the religious obligations which actuate these devoted females to consecrate their lives to the offices of charity. The general affairs of the hospital, exclusive of the interior details, are under the control of a board of directors, acting in conjunction with the Bishop of the Roman Catholic Church in the diocess of Buffalo. By the philanthropic efforts of the latter, the Institution has been created. It is a Catholic Institution, in the denominational sense of that term; but it is, at the same time, Catholic in in a broader sense than when the term is applied to designate a particular denomination of Christians. It is, in other words, in no manner exclusive. It extends relief to the sick of all creeds, and of no creed, making no discrimination on the score of religious, or non-religious professions. Patients are always at liberty to elect their own spiritual counsellors, and every facility is afforded for the gratification of their wishes in this respect. We can testify to a degree of delicacy and unobtrusiveness in this particular, which should satisfy the most fastidious, if not the most uncharitable. Funeral services, in cases of deaths, are also performed by clergymen of any, and all denominations.

Medical and surgical services are furnished by the Medical Faculty of the University of Buffalo, and are gratuitously rendered.

Clinical instruction, as one of the beneficent purposes of public institutions of this character, not least in importance, enters into the enlightened policy with which this hospital is conducted. Students in attendance at the Medical College are permitted to visit the wards in company with the attending surgeon and physician at stated periods, on payment of a small fee, for the benefit of the hospital. Clinical lectures at the bedside, however, are not permitted, and, as we think, with great propriety. To comment at length on the diagnosis, pathology, prognosis, etc., of cases, in the hearing of the patient, is a custom which is revolting to humanity, and should always, in our humble opinion, be interdicted.

EDITOR BUFFALO MED. JOURNAL.

---

This Institution was opened for the reception of patients in August, 1848. Up to the 22d of August, two patients only had been received. Inclusive of these, from the 22d August to the 21st of February, 1849 (inclusive), the number of patients were *one hundred and twenty-one.* The number of patients received during the six months, respectively, are as follows:—

August, 14; Sept. 15; Oct. 18; Nov. 13; Dec. 25; Jan. 22; Feb., to 22d inst., 14; total 121.

Of the whole number, *sixty-one* were charity patients. For *sixty*, compensation was received, generally at the minimum rates prescribed by the regulations of the Hospital.

| | | | | | |
|---|---|---|---|---|---|
| Nativity,— | Americans | 37. | Nativity,— | English | 02. |
| " | Irish | 43. | " | Scotch | 01. |
| " | Germans | 19. | " | French | 01. |
| " | Canadians | 03. | " | Unknown | 13.—121. |

*Sex*—Males, 98; females, 23;—121.

*Civil Condition*—Single, 86; married, 16; widows, 23; widower, 1; unknown, 5;—121.

*Diseases*—Fevers, intermitting, 19; remitting, 2; common continued, or typhoid, 6; typhus, 3. Pulmo-tuberculosis, or phthisis, 7. Bronchitis, 6. Pharyngitis, 2. Pneumonitis, double, 2. Pleuritis, 2. Pericarditis, 1. Laryngitis, 1. Delirium tremens, 2. Enteritis, 1. Acute rheumatism, 2. Dysentery, 1. Dysentery, chronic, 1. Diarrhœa, chronic, 5. Nostalgia, 1. Epilepsy, 3. Anasarca, 1. Neuralgia, 5. Dyspepsia, 1. Melancholia, 1. Prurigo, 1. Psora, 1. Syphilis, primary, 1; secondary, 3; tertiary, 1; consecutive, 1. Varicose veins and ulcers, 4. Ulcer, 2. Ophthalmia, 7. Corneitis and corneal ulcer, 1. Amputation of thigh, 1; of finger, 1. Hernia, 1. Rhinoplastic operation, 1. Onychia maligna, 1. Fractures, compound and comminuted of ankle, 1; of olecranon, 1; of skull, 2; of femur, 4. Contusion and sloughing, 1. Abscess, 1. Lumbar abscess, 1. Burn, 1. Enlarged tonsils, 1. Paraplegia, 2. Chronic inflammation of knee joint, 1. Lithotomy, 1. Impairment of intellect from injury of head, 1. Frostbite of ears and toes, 1.

| | |
|---|---|
| *Results*—Recovered, | 60. |
| Improved | 26. |
| Not Improved, | 01. |
| Absconded, | 01. |
| Died, | 15. |
| Remaining, | 18. |

The diseases in the cases terminating fatally, were as follows:

Chronic diarrhœa of long standing, three cases. In *two* cases, the patients were discharged soldiers who had served in Mexico. In the remaining case, the patient was moribund when brought to the hospital, and died in a few hours after entrance.

Pernicious intermittent, complicating colonitis, *one* case. Patient was picked up in the street, and brought to the hospital. Died second day after entrance.

Fever, continued, 3 cases. In *one* case, death occurred eight days after entrance. In *one*, death occurred sixteen days after entrance, patient entering late in the progress of the disease. In *one*, death occurred 41 days after entrance, from gangrene of lungs, succeeding sloughing of nates.

Pulmo-tuberculosis, 2 cases. Chronic pleuritis, 1 case. Paraplegia and Cystitis, 1 case. Pneumonitis, double. Patient entered in third stage of disease, 1 case. Amputation after injury by rail road cars passing over leg, 1 case. Fracture of skull, of right femur, and left lower extremity below knee, 1 case. Paraplegia from softening of spinal marrow, with Cystitis, 1 case.

The Directors are grateful to Providence, and to a charitable public, for having been enabled, thus far, to carry on an Institution, which, although yet in its infancy, they have reason to believe has saved the lives of many. They hope to be able to give relief to still greater numbers, when they have completed the works already commenced. The comforts of religion, when asked for, have been administered by Roman Catholic, or by Protestant clergymen.

Buffalo, February, 1849.

